# Restorative Dental Resin Functionalized with Calcium Methacrylate with a Hydroxyapatite Remineralization Capacity

**DOI:** 10.3390/ma16196497

**Published:** 2023-09-29

**Authors:** Xin Zhang, Yuxuan Zhang, Ying Li, Xiaoming Wang, Xueqin Zhang

**Affiliations:** 1 College of Chemistry and Materials Engineering, Beijing Technology and Business University, Beijing 100048, China; 2 FuYang Sineva Materials Technology Co., Ltd., Beijing 100176, China; zhangyuxuan@sineva.com.cn; 3 Shuozhou Comprehensive Inspection and Testing Center, Shuozhou 036000, China

**Keywords:** dentistry, dental materials, dental resin, photopolymerization, hydroxyapatite, calcium methacrylate, remineralization

## Abstract

The ability of dental materials to induce the mineralization of enamel like hydroxyapatite (HA) is of great importance. In this article, a novel kind of dental restorative material characterized by a mineralization ability was fabricated by photopolymerization. Calcium methacrylate (CMA) was introduced into the classical bisphenol A-glycidyl methacrylate (Bis-GMA) and triethylene glycol dimethacrylate (TEGDMA) dental resin formulation. This functional dental resin (BTCM) was calcium-rich and can be prepared simply by one-step photopolymerization. The influence of CMA on the photopolymerization kinetics, the dental resin’s mechanical properties, and its capacity to induce dynamic in situ HA mineralization were examined. Real-time FTIR, compression modulus, scanning electron microscopy, X-ray spectroscopy, MTT assay, and cell attachment test were carried out. The obtained data were analyzed for statistical significance using analysis of variance (ANOVA). Double bond conversion could be completed in less than 300 s, while the compression modulus of BTCM decreased with the increase in CMA content (30 wt%, 40 wt%, and 50 wt%). After being soaked in Ca(NO_3_)_2_ and Na_2_HPO_4_ solutions alternatively, dense HA crystals were found on the surface of the dental resin which contained CMA. The amount of HA increased with the increase in CMA content. The MTT results indicated that BTCM possesses good biocompatibility, while the cell adhesion and proliferation investigation demonstrated that L929 cells can adhere and proliferate well on the surface of BTM. Thus, our approach provides a straightforward, cost-effective, and environmentally friendly solution that has the potential for immediate clinical use.

## 1. Introduction

Enamel serves as the outermost protective layer of teeth, primarily comprising inorganic minerals, with hydroxyapatite (HA) being the predominant component, making up approximately 96% to 98% of its composition [1,2]. Enamel mineralization is a complex extracellular process regulated by matrix proteins, especially enamel proteins, which control the nucleation, growth, and self-assembly of crystals [3]. However, enamel is vulnerable to demineralization due to acid erosion or mechanical wear, leading to enamel loss and the formation of cavities [4]. Mature enamel is non-vital tissue, which means that it lacks the ability for rapid self-repair once cavities have formed [5]. Given the importance of enamel in tooth protection and oral health, the repair of enamel defects is of significant importance. Enamel can be effectively restored using biomimetic repair strategies, which focus on chemical consistency and structural restoration. The traditional dental restoration strategy, which employs materials such as metal [6,7], ceramic [8], and composite resin [9], primarily follows a dislocated repair approach. In contrast, biomimetic mineralization repair represents an in situ repair strategy, allowing the regeneration of calcium phosphate crystals in the mineral-deficient area [10,11,12]. Incorporating the component with the ability to bind with Ca^2+^ or PO_4_^3−^ into the dental restorative materials can enhance the remineralization capacity of enamel lesions caused by dental caries. Moreover, releasing Ca^2+^ and PO_4_^3−^ around the carious region to increase the oversaturation of HA can lead to more Ca^2+^ and PO_4_^3−^ from saliva being deposited onto the carious lesion [13,14]. Historically, fluoride was the first and has long been used as a highly effective agent for inhibiting the formation of dental caries and promoting enamel remineralization [15,16]. Fluoride can directly react with HA crystals in the solution to form fluoroapatite (FHAP) or fluorapatite (FAP) [17]. Recently, various biomimetic systems containing ACP (amorphous calcium phosphate) nanoparticles which significantly enhance enamel remineralization have been developed [18]. There has been significant research focused on commercialized casein phosphopeptide-ACP (CPP-ACP) [19,20,21]. CPP-ACP can promote enamel remineralization, and its synergistic effect with fluoride further enhances the remineralization process [22]. In addition, biomimetic HA is a highly effective enamel repair material due to its similar chemical composition to enamel [23,24]. Clinical trials demonstrated that biomimetic HA paste can effectively reduce dental sensitivity and improve enamel integrity [25]. In addition, dental resin composites incorporated with one or a variety of reinforcing fillers such as bioactive glass [26,27], zeolites [28,29,30], HA [31,32], ACC [33,34], silica [35], calcium fluoride [36], CPP-ACP [14,37] and so on, have found widespread use in repairing decayed teeth due to their enhanced HA regeneration ability, substantial mechanical strength, excellent aesthetic results, minimal health concerns, and ease of handling properties [38,39,40]. A typical composition for dental resin composites includes a resin matrix composed of monomers such as bisphenol A-glycidyl methacrylate (Bis-GMA) and triethylene glycol dimethacrylate (TEGDMA) [41]. Liu et al. [42] developed a poly (Bis-GMA)-grafted HA-whisker-reinforced Bis-GMA/TEGDMA dental resin with a reduced volume shrinkage and enhanced flexural strength. Sandomierski et al. [43] fabricated Bis-GMA/TEGDMA filled with a calcium montmorillonite filler coated with HA, demonstrating its capacity to promote HA mineralization. Qin et al. [31] prepared a salinized HA nanofiber filler loaded with erythromycin (s-HAFs@EM) to reinforce Bis-GMA/TEGDMA resin. The addition of s-HAFs@EM imparted the dental composite with an excellent antibacterial activity and HA remineralization capacity. Jardim et al. [44] incorporated HA nanoparticles (HANPs) into Bis-GMA/TEGDMA dental resin matrix. This composite was capable of releasing Ca^2+^ and PO_4_^3−^ ions, thereby enhancing the remineralization ability.

**Figure 1:**
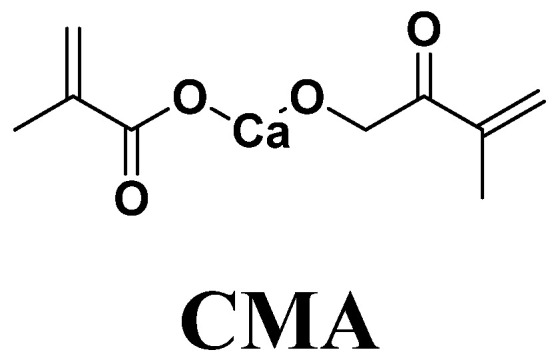
Chemical structure of CMA.

Nonetheless, the complex oral environment and the impact from chewing can result in irrevocable or enduring loss of fillers in the restorative composite, further reducing their ability to induce HA regeneration. Calcium methacrylate (CMA, Figure 1), a bifunctional monomer and the calcium salt of methacrylic acid, can also be employed as a monomer for photopolymerization [45]. The photopolymerization product of CMA can not only serve as a binding site for PO_4_^3−^ in the oral environment but also release Ca^2+^, facilitating the deposition of HA on its surface. When CMA is copolymerized within the Bis-GMA/TEGDMA resin system, the resulting polymer gains the capability to induce HA remineralization. Even if the surface is abraded, it retains the ability to promote HA remineralization because CMA is copolymerized within the resin network.

Thus, in this work, a novel dental restorative material characterized by a mineralization ability was fabricated by photopolymerization. CMA was introduced into the classical Bis-GMA/TEGDMA dental resin formulation. This functional dental resin Bis-GMA/TEGDMA/CMA (BTCM) was calcium-rich and could be prepared simply by one-step photopolymerization. Through copolymerization with CMA, the resulting BTCM could induce the mineralization of a dense HA layer and could continue to stimulate the in situ generation of HA even if the surface layer was damaged. The primary purpose of this work is to endow dental restorative resin with the ability to regenerate HA and achieve self-repair in an oral environment by constructing a calcium-rich 3D polymer network structure. Another objective is to establish a simple and controllable method for the production of dental resin, facilitating scalable applications.

## 2. Materials and Methods

### 2.1. Materials

Bisphenol A-glycidyl methacrylate (Bis-GMA) and triethylene glycol dimethacrylate (TEGDMA) were kindly supplied by Sartomer Company (Warrington, PA, USA). Calcium methacrylate (CMA) and methacrylic acid (MAA) were purchased from Sigma-Aldrich (St. Louis, MO, USA). Camphorquinon (CQ) was obtained from Runtec Co., Ltd. (Jintan, Jiangsu, China). Ethyl 4-dimethylaminobenzoate (EDAB), calcium nitrate anhydrous (Ca(NO_3_)_2_•4H_2_O, analytical reagent), sodium dihydrogen phosphate (Na_2_HPO_4_•12H_2_O, analytical reagent), hydrochloric acid (HCl, 37.5%), and sodium hydroxide (NaOH, analytical reagent) were purchased from Sinopharm Chemical Reagent Co., Ltd. (Shanghai, China). 3-(4,5-dimethylthiazol-2-yl)-2,5-diphenyltetrazolium bromide tetrazole (MTT), Dulbecco’s modified eagle medium (DMEM), fetal calf serum (FBS), and phosphate-buffered saline (PBS) were purchased from Sigma-Aldrich (St. Louis, MO, USA). Dimethyl sulfoxide (DMSO) and toluene were purchased from InnoChem Science & Technology Co., Ltd. (Beijing, China).

### 2.2. Methods

#### 2.2.1. The Fabrication of Calcium Poly(methyl methacrylate) (BTCM)-Based Dental Material

**Figure 2:**
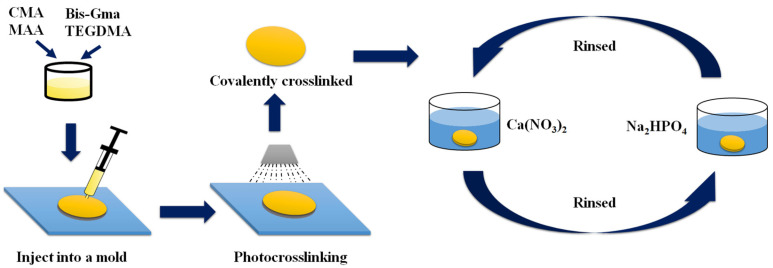
Diagram illustrating the process of mineralization of BTCM.

Bis-GMA, TEGDMA, CMA, MAA, and the photoinitiation system (CQ and EDAB) were mixed in specific ratios as indicated in Table 1. Notably, the weight ratios of Bis-GMA to TEGDMA and CQ to EDAB were maintained at 7:3 and 1:1, respectively. The mixture was intensely stirred for 20 min and then subjected to 15 min of sonication to eliminate any air inside the container. The resulting mixture was subsequently added into a polytetrafluoroethylene (PTFE) cylindrical mold. Photocrosslinking was carried out for 10 min by using an LED light with an emission wavelength at 460 nm, and the light intensity was set to 50 mW cm^−2^ [46]. The sample BTCM-1, without adding any CMA, was used as the control group. The preparation steps are illustrated in Figure 2.

#### 2.2.2. The Mineralization of BTCM

The remineralization of HA induced by BTCM was achieved via alternative soaking process (ASP) [47]. A Ca(NO_3_)_2_ solution of 0.5 mol L^−1^ and a Na_2_HPO_4_ solution of 0.3 mol L^−1^ were prepared by dissolving the calculated amount of Ca(NO_3_)_2_ and Na_2_HPO_4_ in deionized water, respectively. By using 1 mol L^−1^ NaOH, the pH of the Ca(NO_3_)_2_ and Na_2_HPO_4_ solutions was adjusted to 10. The photopolymerized BTCMs were preconditioned in deionized water for 60 s and then soaked in the Ca(NO_3_)_2_ solution for 4 h. Afterward, the samples were taken out and rinsed with deionized water for 60 s before being soaked in the Na_2_HPO_4_ solution for 4 h. This entire process constituted one cycle of ASP (Figure 2). Three cycles of ASP were performed for the mineralization of each BTCM sample. Following these three cycles of ASP, BTCM samples were washed with deionized water and placed in a vacuum dryer at 50 °C for 24 h.

#### 2.2.3. Remineralization of HA on the Abraded BTCM Matrixes

To simulate the abrasion of the HA layer, the surface of each BTCM matrix, which was already mineralized with HA, were polished using sandpaper. After polishing, the mineralized HA was removed, and the inner polymer was exposed. All polished BTCM samples were then set to three cycles of ASP [47] to induce the remineralization of HA (Figure 2). Each cycle lasted for 8 h. The BTCM samples obtained from three cycles of ASP were washed with deionized water and placed in a vacuum dryer at 50 °C for 24 h.

### 2.3. Characterization

#### 2.3.1. Photopolymerization Kinetics

The kinetics analysis of photopolymerization reactions was investigated using Realtime-FTIR (Nicolet 5700, Nicolet Instrument Corp., Madison, WI, USA), equipped with an MCT/A KBr detector [48,49]. The BTCM mixtures with varying compositions were applied between two layers of KBr salt plates using a capillary and placed on a real-time infrared horizontal sample stage. An LED light with an emission wavelength of 460 nm was utilized as the light source for photopolymerization. The infrared absorption peak of the C=C double bonds in CMA was observed around 1640 cm^−1^, while the absorption peak of the C=C double bonds in Bis-GMA/TEGDMA was observed around 1680 cm^−1^. Information about the progress of the reaction was obtained by monitoring changes in its peak area. The final conversion rate of the polymerization reaction was calculated using Equation (1), with data processing carried out using the OMNIC 7.5 infrared software (Thermo Fisher Scientific, Wilmington, MA, USA) and the ORIGIN 8.0 (OriginLab Corporation, Norwood, MA, USA) data processing software.

(1) $$DC(\%)=\frac{A_{0}-A_{t}}{A_{0}}\;100\%\;$$

where *DC* is the conversion of C=C double bonds at *t* time, *A*_0_ represents the peak area of C=C double bonds before photopolymerization, and *A_t_* represents the peak area of C=C double bonds after photopolymerization at time *t*.

#### 2.3.2. Thermogravimetric Analysis

The thermal stability of the BTCM dental resin with different CMA concentrations was investigated using a TA Q500 thermogravimetric analyzer (TA Instruments, New Castle, DE, USA) [50]. The BTCM samples were ground into a powder, and 5 mg was weighed and placed in a platinum sample pan for testing. Under a nitrogen atmosphere, the sample was heated from room temperature to 800 °C at a ramp rate of 10 °C/min. The nitrogen flow rate was set to 40 mL/min.

#### 2.3.3. X-ray Diffraction Analysis (XRD)

The crystallinity of the crystals obtained from BTCM after three cycles of ASP was examined by an X-ray diffraction analysis. An X-ray diffractometer with Cu Kα radiation (λ = 0.154 nm), a 40 kV voltage, and a 40 mA current (Rigaku D/Max2500VB^2+^/Pc diffract meter, Rigaku Company, Tokyo, Japan), was used [50]. The scanning range was from 10° to 70°, with a scanning speed of 5°/min.

#### 2.3.4. Mechanical Compression Test

The mechanical properties of the BTCM samples were investigated using an Instron 4505 universal materials testing machine (Instron, High Wycombe, UK) with a 10 kN load cell [51]. The samples were cylindrical columns with a diameter of 8 mm and a depth of 12 mm. The Instron testing machine’s probe was programmed to descend at a rate of 5 mm/min, and the load was applied until the specimen’s height was reduced by 30%. Testing was conducted using three samples for each composition.

#### 2.3.5. Scanning Electron Microscopy (SEM)

The morphology of the BTCM samples, before and after three cycles of ASP, was observed by utilizing a Hitachi S-4700 (Hitachi, Tokyo, Japan) scanning electron microscope [51]. The BTCM samples were cut into the required shapes and placed on the sample stage, followed by gold coating before observation.

#### 2.3.6. In Vitro Cell Cytotoxicity

The biocompatibility of the BTCM dental material was assessed through MTT cytotoxicity assays [46]. Prior to cell incubation, the BTCM slices were immersed in ultrapure water and ethanol for 18 h, respectively, to remove monomers and residual small molecules. The samples, which had undergone ultrapure water and ethanol soaking, were then subjected to high-pressure steam sterilization for sterilization and disinfection.

L929 mouse fibroblast cells were seeded in a sterile 96-well plate, and the density was 1 × 10^4^ cells per well. After 24 h of culturing at 37 °C in an incubator with a humid atmosphere, 5% carbon dioxide, and a temperature of 37 °C, 100 μL of DMEM cell suspension was added to each well of the 96-well plate. The prepared BTCM samples were subsequently put into the confluent layer of L929 cells and further incubated under the same conditions. A PBS solution of 5 mg/mL MTT was prepared, and 20 μL of this solution was added to the 96-well plate after 24 h, 48 h, and 72 h of incubation, followed by another 4 h of incubation. Then, DMSO was added to the 96-well plate, and the formed MTT was dissolved by DMSO. Once the MTT had dissolved, the optical density (OD) at 595 nm was examined by using an enzyme-linked immunosorbent assay (ELISA) reader (Multiskan FC instrument, Thermo Fisher Scientific, Waltham, MA, USA), for each time point (24 h, 48 h, and 72 h). The biocompatibility of BTCM was determined by comparing the OD values of the sample group with those of the negative and positive control groups. In this context, toluene served as the positive control, while DMEM with 0.1% DMSO served as the negative control. Testing was conducted using three samples for each composition.

#### 2.3.7. Cell Adhesion and Proliferation Investigation

The adhesion and proliferation of L929 cells on the BTCM surfaces were investigated using SEM [51]. Firstly, the BTCM samples were sterilized by autoclaving, and then these samples were affixed onto glass slides and placed in sterilized 24-well plates. Sterilized PBS buffer was added to the 24-well plate, followed by the addition of 1 mL of L929 cell suspension at a density of 1.5 × 10^4^ cells mL^−1^. After incubation in a humidified atmosphere with 5% carbon dioxide at 37 °C for 24 h, the cell-populated samples were removed and washed with PBS solution. The cells on the BTCM surfaces were fixed using a 2.5% glutaraldehyde solution and underwent a gradual dehydration process. After drying, the BTCM samples were gold-coated and then examined using SEM.

#### 2.3.8. Statistical Analysis

IBM SPSS 25 (IBM Corp., Armonk, NY, USA) was used to analyze variance (ANOVA) for statistical analysis. All quantitative data are denoted in the form of “mean ± standard deviation”. The compression test and MTT test were repeated three times independently, and a *p*-value <0.05 was considered statistically significant.

## 3. Results and Discussion

### 3.1. Photopolymerization Kinetics

**Figure 3:**
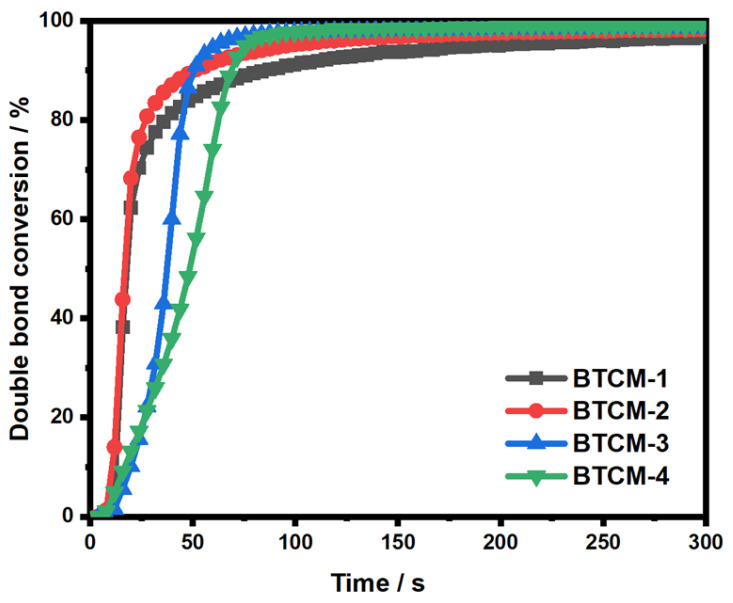
Photopolymerization kinetics of BTCM with different compositions.

The plots showing the double bond conversion (DC) versus irradiation time of Bis-GMA and TEGDMA incorporating different concentrations of CMA, irradiated by 460 nm wavelength light with an intensity of 50 mW cm^−2^, are displayed in Figure 3. As shown in Figure 3, the DC of BTCM-1, BTCM-2, BTCM-3, and BTCM-4 all reached approximately 100% within 300 s (Table 2). This indicates that the double bonds in all samples were completely polymerized, forming C-C covalent bonds, ensuring the absence of small monomer molecule residues in the system. This is essential for BTCM’s use as a dental material. With the increase in CMA concentration, the photopolymerization speed decreased, and the induction time of the photopolymerization system increased. This can be attributed to the positive charge of Ca^2+^ in CMA, which reduces the electron density in the double bond of CMA, thereby reducing the reactivity of the C=C double bond and leading to a decrease in the photopolymerization rate and an increase in the induction time. It is worth noting that the photopolymerization rate increased when 30% CMA (BTCM-2) was added to the resin system, while it decreased when 40% and 50% CMA (BTCM-3 and BTCM-4) were added to the resin system. This variation in the photopolymerization rate can be assigned to the joint effect of CMA and MAA on the viscosity of the resin mixture. By adding CMA and MAA into the formulation, the viscosity of the photopolymerization system decreased, leading to a higher molecule mobility and an increased reaction rate. However, excessive CMA in the formulation led to a reduction in the reaction rate, primarily due to the decreased electron density of the C=C bond.

### 3.2. Thermogravimetric Analysis (TGA)

**Figure 4:**
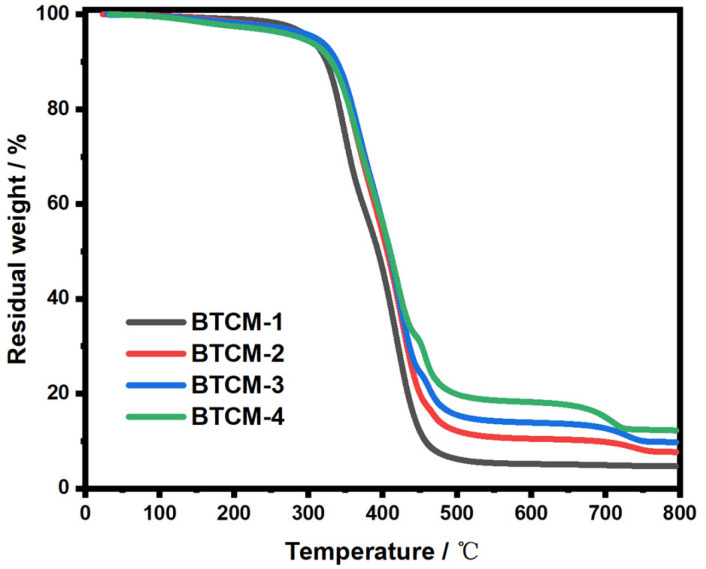
TGA curves of BTCM-1, BTCM-2, BTCM-3, and BTCM-4, respectively.

The thermal degradation properties of the BTCM samples were explored through TGA. Figure 4 shows the TG curves for BTCM samples with different compositions. BTCM-1 exhibited a residual mass of 4.6%, primarily attributable to the remaining carbon content, while BTCM-2, BTCM-3, and BTCM-4 exhibited a residual mass of 7.7%, 10.1%, and 12.3% at 800 °C, respectively (Table 3). The remaining weight represented both the calcium and residual carbon content. The residual mass increased with the increase in CMA concentration. During the heating process, the copolymer breaks down and converts into small molecules, such as carbon dioxide, alkanes, and alkenes, which exit the reaction furnace at high temperatures. Simultaneously, the calcium present in the system undergoes transformation into CaCO_3_ and ultimately decomposes into CaO, which remains in the heating furnace. A higher calcium content in the system leads to a higher final residual mass after heating.

### 3.3. Compression Modulus Analysis

**Figure 5:**
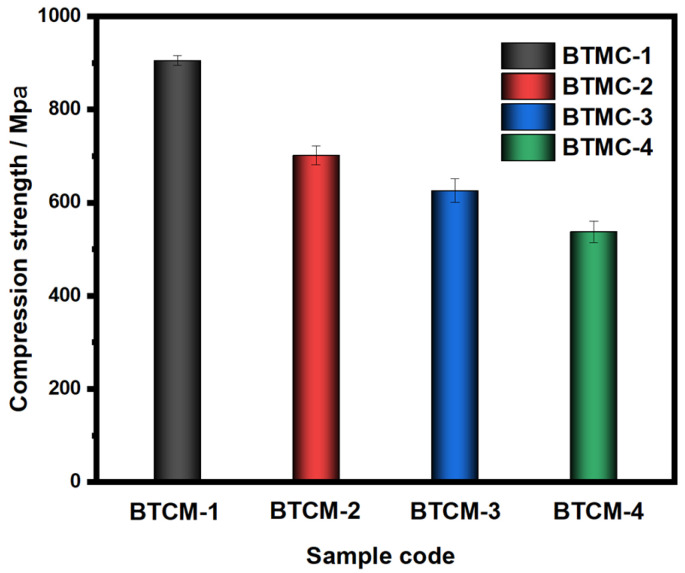
Compression moduli of BTCM samples with different compositions. The error bars represent the standard deviation of three replicates.

The mechanical characteristics of dental materials are crucial in the long-term durability of biomaterials. The compression moduli of the BTCM samples are depicted in Figure 5 and Table 4. As can be seen, the compression moduli of BTCM-1, BTCM-2, BTCM-3, and BTCM-4 were found to be 905.36 ± 10.68, 701.58 ± 20.13, 625.71 ± 25.69, and 537.43 ± 23.45 MPa (Table 4), respectively. The samples contained CMA showed statistically significant differences from BTCM-1 (*p* = 0.000 < 0.005). This indicates that the presence of CMA in the system has a substantial impact on the mechanical properties of BTCM. As the concentration of CMA increased, the compression modulus decreased. Since both CMA and MAA are present in the photopolymerization system, the mechanical properties of their polymerization product were significantly lower than those of BTCM-1, resulting in a decrease in the compression modulus.

### 3.4. X-ray Diffraction (XRD) Analysis

**Figure 6:**
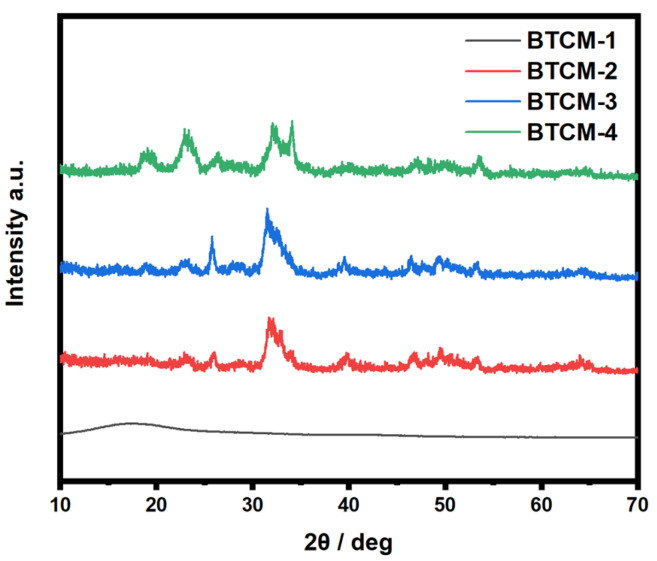
XRD patterns of the BTCM samples after three cycles of ASP.

Figure 6 displays the XRD patterns of the BTCM dental materials after three cycles of ASP. BTCM-1 exhibited a broad diffraction peak between 10° and 25°, indicating that BTCM-1 was amorphous and lacked bioactivity to induce the mineralization of HA. In contrast, BTCM-2, BTCM-3, and BTCM-4 all exhibited characteristic peaks of HA after three cycles of ASP, appearing at 2θ = 25.7°, 31.56°, 39.48°, 46.42°, and 53.38°, corresponding to the (002), (211), (310), (203), and (004) diffraction planes of HA (JCPDS #9-432), respectively [52,53]. Furthermore, BTCM-3 and BTCM-4 demonstrated better bioactivity, as mineralization of HA could be achieved within 24 h. Furthermore, BTCM-3 and BTCM-4 displayed diffraction peaks with higher intensities than BTCM-2, which indicated that the HA content was higher on BTCM-3 and BTCM-4. This is attributed to the higher calcium content in the polymerized materials. The greater the calcium content, the more HA can be generated.

**Figure 7:**
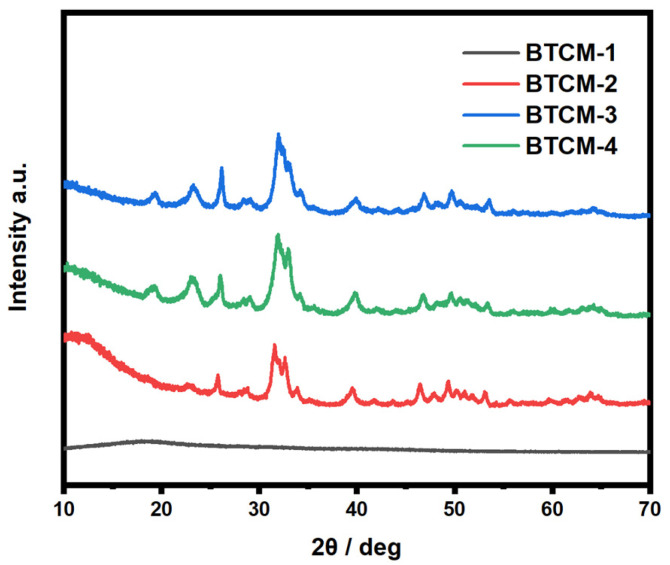
XRD patterns of the polished BTCM samples after 3 cycles of ASP.

To investigate the ability of BTCM to induce a dynamic HA regeneration, the surface of BTCM, which was covered with a layer of HA, was polished to expose the polymer once again, in order to simulate the damage or erosion of enamel. Subsequently, another three cycles of ASP were conducted, and the XRD patterns of BTCM after the ASP experiments are shown in Figure 7. The polished BTCM samples subjected to three new cycles of ASP exhibited similar crystallinity to the BTCM samples after the first three cycles of ASP. BTCM-1, once again, displayed a broad peak at 2θ = 10–25°, indicating its amorphous nature and low bioactivity. BTCM-2, BTCM-3, and BTCM-4 all exhibited diffraction peaks identical to those observed in the first round of ASP, specifically at 2θ = 25.7°, 31.56°, 39.48°, 46.42°, and 53.38°, corresponding to the (002), (211), (310), (203), and (004) diffraction planes of HA (JCPDS #9-432). The results indicate that the prepared BTCM dental materials possess a robust ability to stimulate HA regeneration, suggesting their potential in clinical utility as dental materials.

### 3.5. Morphology Analysis by Scanning Electron Microscopy (SEM)

**Figure 8:**
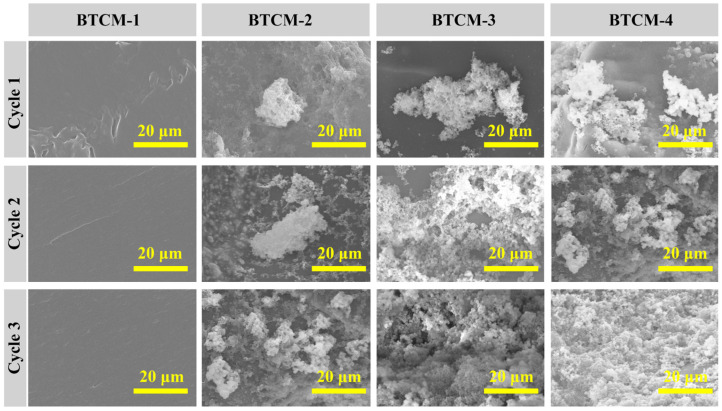
SEM images showing the evolution of the HA layer on BTCM samples with different compositions after 3 cycles of mineralization (scale bar: 20 μm).

The morphology of the BTCM samples after three cycles of ASP was investigated by SEM, and the corresponding images are shown in Figure 8. As can be seen, with the increase in CMA concentration, a crystal growth evolution from spheroidal sediment to flocculent crystal sediment on the surface of BTCM can be observed. After three cycles of ASP, the surface of BTCM-1 appeared flat and smooth, which is consistent with the XRD patterns. This observation suggests that BTCM cannot induce the mineralization of HA due to the absence of binding sites for Ca^2+^ or PO_4_^3−^, which is required for mineralization. BTCM-2, BTCM-3, and BTCM-4 all exhibited strong bioactivity. After one cycle of mineralization, BTCM-2 exhibited spheroidal sediment on its surface. Meanwhile, BTCM-3 and BTCM-4 exhibited flocculent sediment composed of spheroidal crystals on their surfaces. After three cycles of mineralization, densely packed clusters of spheroids were found on the surfaces of BTCM-2, BTCM-3, and BTCM-4, indicating the excellent bioactivity of BTCM.

**Figure 9:**
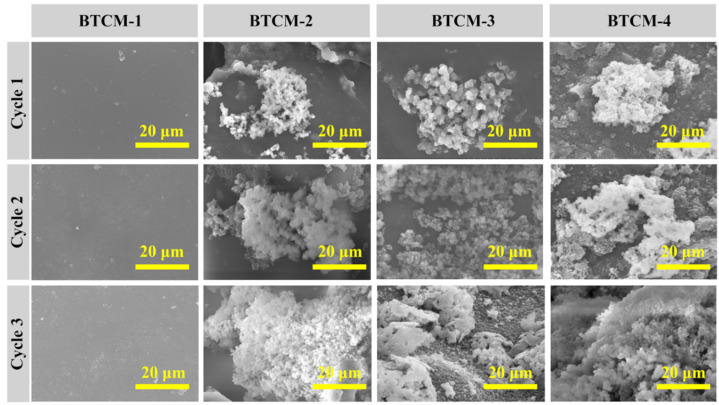
SEM images showing the evolution of the HA layer on BTCM samples with different compositions after 3 cycles of remineralization (scale bar: 20 μm).

To further investigate the ability of BTCM in inducing dynamic HA regeneration, the mineralized BTCM samples were polished and set to three new cycles of ASP and the mineralization results are shown in Figure 9. BTCM-1 showed no capacity to induce the mineralization of HA, while BTCM-2, BTCM-3, and BTCM-4 exhibited a similar trend to that observed during the first three cycles of mineralization. After the first cycle, spheroids of crystals appeared on the surfaces of BTCM-2, BTCM-3, and BTCM-4. As time progressed, thick layers of clustered spheroidal crystals developed on the surfaces of BTCM-2, BTCM-3, and BTCM-4, once again indicating the ability of the BTCM materials to induce a dynamic mineralization of HA on their surfaces. These results indicate that the calcium-rich BTCM dental restorative resin can effectively induce a dynamic and in situ remineralization of HA.

**Figure 10:**
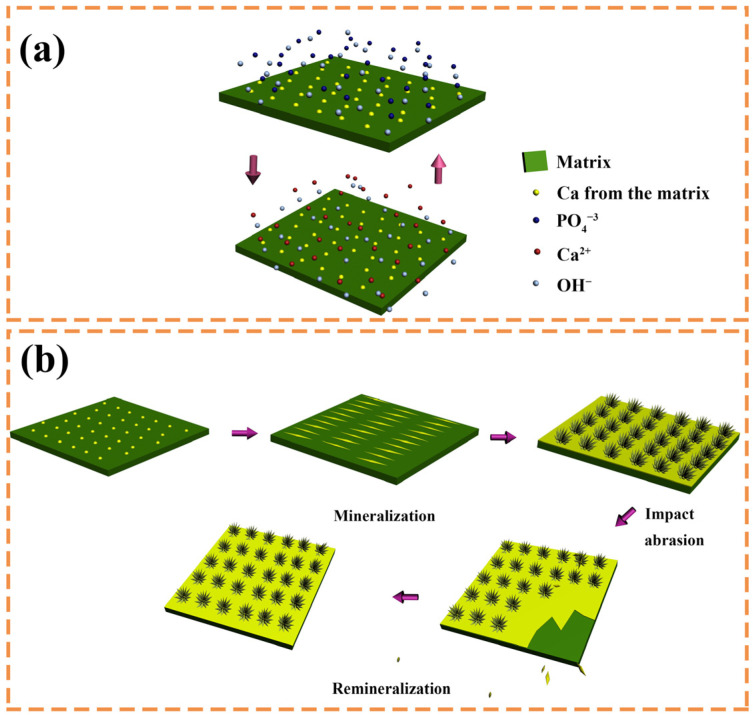
The mechanism of the dynamic mineralization of HA on BTCM samples. (**a**) PO_4_^3−^ and Ca^2+^ accumulate around the BTCM matrix and lead to the mineralization of HA during the ASP process; (**b**) Abraded HA-rich matrix could again induce HA mineralization.

Based on the XRD patterns and SEM images of the mineralized BTCM samples, an underlying HA growth mechanism could be inferred. Within the BTCM matrix, there are large quantities of calcium ions and carboxyl groups that serve as nucleation sites and binding sites for Ca^2+^ and PO_4_^3−^ during the mineralization of HA. In the early stages, when BTCM was immersed in a solution containing PO_4_^3−^, the PO_4_^3−^ ions rapidly accumulate around the positively charged Ca^2+^ ions, forming a locally supersaturated calcium phosphate solution (Figure 10a). When the system’s Gibbs free energy decreased to a critical value, nucleation of crystals occurred. Subsequently, when PO_4_^3−^-bounded BTCM was immersed in Ca(NO_3_)_2_ solution, the substrate bound with the Ca^2+^ in the solution to generate amorphous calcium phosphate precursors. After three cycles of mineralization, more minerals, which were identified as HA (Figure 6 and Figure 7), were generated. Moreover, when the HA-rich matrix was abraded and the former mineralized HA was removed, the Ca^2+^- and carboxyl group-rich matrix could once again induce HA mineralization. Consequently, the matrix is covered by HA after immersion in the appropriate environment (Figure 9 and Figure 10b). Enamel demineralization refers to the dissolution of the HA mineral, hydroxyapatite mineral, in the teeth under acidic conditions in the oral cavity, while enamel remineralization refers to the reprecipitation of calcium, phosphate, and other mineral ions on the surface of the teeth in situations of normal or localized demineralization. Enamel in a healthy oral environment with the presence of saliva is relatively stable, and demineralization and remineralization are continuous and alternating processes that occur [54]. Thus, BTCM can simulate the dynamic mineralization and demineralization processes that occur in the oral cavity, making it a potential dental material capable of inducing the dynamic mineralization of HA in the oral environment.

### 3.6. MTT Toxicity Assay

**Figure 11:**
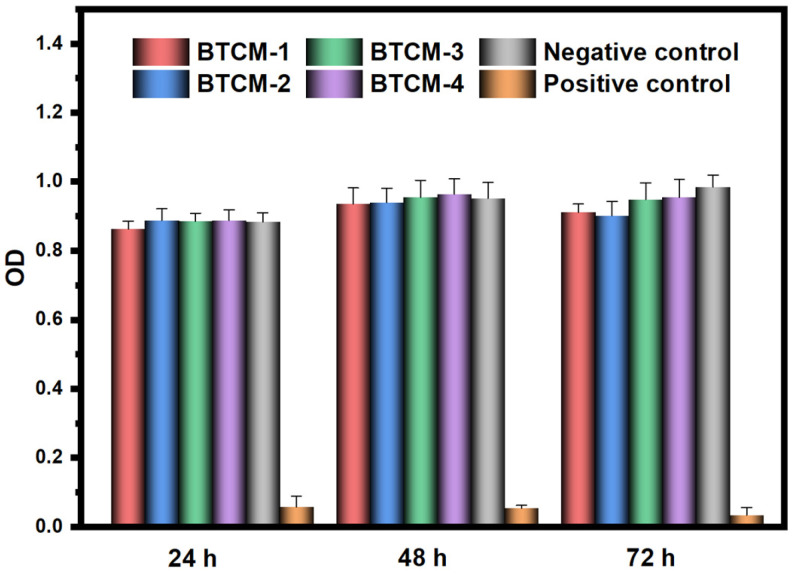
Cell viability study of different BTCM dental resins. The error bars represent the standard deviation of three samples.

An ideal dental material should not release toxic substances or induce harmful reactions within the human oral cavity. An MTT cytotoxicity assay was employed to investigate the toxicity of the BTCM dental materials. In this test, the viability of L929 cells was assessed after 24 h, 48 h, and 72 h of in vitro culture to evaluate the material for potential toxicity, and the results are shown in Figure 11 and Table 5. After 24 h of cultivation, the viability of all L929 cells was high, and there was no significant difference (*p* > 0.05) between BTCM-1, BTCM-2, BTCM-3, BTCM-4, and the negative control group, while there were statistically differences between the BTCM samples and the negative control group (*p* = 0.000 < 0.005). After 48 h, the OD values for all four groups had increased, suggesting cell proliferation during this period. The absorbance values of the four BTCM samples were not significantly different from the negative control group. After 72 h of cultivation, the L929 cell viability on the surfaces of BTCM-1, BTCM-2, BTCM-3, and BTCM-4 slightly increased, suggesting that BTCM was non-toxic to L929 cells and was unlikely to pose toxicity concerns for oral tissues upon implantation. Therefore, BTCM dental materials demonstrate good biocompatibility and hold promise for clinical applications.

### 3.7. Cell Adhesion and Proliferation Analysis

**Figure 12:**
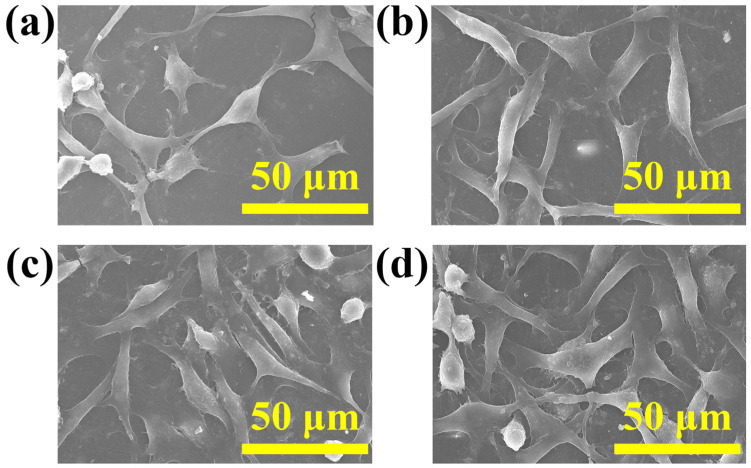
The morphology of L929 cells attached on BTCM: (**a**) BTCM-1; (**b**) BTCM-2; (**c**) BTCM-3; and (**d**) BTCM-4 (scale bar: 50 μm).

The SEM photos in Figure 12 depict L929 cells adhering to BTCM samples prepared with varying concentrations of CMA following 72 h of cultivation. As can be seen, the L929 cells were evenly distributed on the surfaces of the BTCM samples, and the cell population was higher on the BTCM samples with higher CMA content. Notably, on the surfaces of BTCM-3 and BTCM-4, L929 cells spread completely across the material surfaces. Moreover, cells at various growth stages were observed. These results indicated that as the CMA content increased, the biocompatibility of BTCM improved. In conclusion, BTCM exhibited excellent cell adhesion properties. Cross-linked CMA showed no toxicity to cells, and with the increase in calcium content in the system, the biocompatibility also increased. Therefore, the BTCM dental material is non-toxic and demonstrates exceptional biocompatibility.

## 4. Discussion

The self-repair ability of tooth enamel is quite limited when the enamel is damaged by acid erosion or cavities. As enamel plays a vital role in the protection of oral health, the significance of enamel restoration is substantial. Integrating components with photoactivity and remineralization properties into the classical dental resin Bis-GMA/TEGDMA formulation is an effective strategy for improving the remineralization property of dental restorative resin. Former studies usually realized the mineralization of Bis-GMA/TEGDMA dental resin by incorporating fillers or coatings with a remineralization ability to the resin formulation [9,55,56]. In this study, a bifunctional monomer called CMA, which is the calcium salt of methacrylic acid, was incorporated into the Bis-GMA/TEGDMA dental resin formulation to develop a bioactive dental restorative resin. The photopolymerization product BTCM was calcium-rich and demonstrated an excellent HA regeneration ability.

Photopolymerization efficiency and double-bond conversion are two important factors for dental resin. Although the addition of CMA led to a decrease in photopolymerization speed to some extent due to the lower reactivity of CMA, the photopolymerization can still be completed in less than 300 s, demonstrating the high photoactivity of the BTCM dental resin. However, the double-bond conversion of BTCM slightly increased with the increase in CMA. This is caused by the decreased viscosity of the formulation, which can lead to a higher mobility of the molecules, allowing more double bonds to polymerize. Tian et al. [57] incorporated nano fibrillar silicate (FS) in Bis-GMA/TEGDMA dental resin which also exhibited a high double-bond conversion and a high photopolymerization speed.

Although the compression modulus decreased with an increase in CMA content, BTCM still exhibited sufficient mechanical properties for dental restoration applications [58,59]. To obtain a transparent mixture of Bis-GMA/TEGDMA/CMA when prepare the BTCM formulation, MAA has to be added into the formulation so that CMA can be dissolved completely. The photopolymerization product of MAA is often used as a hydrogel [60], drug carrier [61], polyelectrolyte [62] and so on. Thus, the incorporation of MAA can lead to the decrease in the compression modulus of BTCM. In a study conducted by Liu et al. [42], Bis-GMA and TEGDMA were grafted onto the surface of HA whiskers. These whiskers were subsequently utilized as fillers in Bis-GMA/TEGDMA dental restorative resin composites, resulting in an enhanced compression modulus.

Combined with the XRD results, the mineralization findings showed that the BTCM restorative dental resin exhibited good bioactivity. When immersed alternately in Ca(NO3)_2_ and Na_2_HPO_4_ solutions, the surface of the BTCM samples became covered with HA crystals, and the layer became denser with increasing immersion time. This is because the surface of BTCM contains large amounts of calcium and carboxyl groups which can bind with PO_4_^3−^ and Ca^2+^ to generate HA crystals. The CMA content has a significant influence on the bioactivity of BTCM. With an increase in CMA concentration, the mineralized HA layer on the surface of BTCM is thicker due to the increased binding sites for Ca^2+^ and PO_4_^3−^. Enamel primarily consists of HA crystals with complex hierarchical structure [63,64]. Within enamel, HA crystals run parallel to each other along the long axis, forming enamel rods that interlock internally. This unique arrangement is vital for enhancing enamel’s mechanical strength and resistance to fissures. As a result, it is also important to control the structure of the mineralized HA crystals. However, the HA crystals in our work were not well arranged. Shao [65] designed a material composed of calcium phosphate ion clusters (CPICs) that can result in a precursor layer with a continuous mineralization interface, inducing epitaxial crystal growth of enamel HA, which mimics the biomineralization crystal-precursor frontier of enamel development. Furthermore, in combination with the cell adhesion results, all BTCM samples demonstrated excellent bioactivity, as evidenced by the high viability of L929 cells cultivated on their surfaces. There were no statistically significant differences between the BTCM samples and the positive control group.

## 5. Conclusions

CMA-functionalized bis-GMA/TEGDMA dental resin with the ability to induce the remineralization of HA was fabricated in this work. The calcium-rich restorative dental material with good bioactivity could be obtained by photopolymerization. The photopolymerization kinetics were investigated by real-time FTIR. The photopolymerization rate increased first with the increase in CMA concentration, but then decreased with higher CMA concentrations. The compression test results indicated a negative impact of CMA on the compression modulus of BTCM. The bioactivity which showed the HA mineralization ability of BTCM was confirmed through SEM and XRD patterns. Calcium within the BTCM matrix played a significant role in this bioactivity. With three cycles of ASP, BTCM-2, BTCM-3, and BTCM-4 all exhibited densely clustered spheroids of HA on their surfaces. The mineralization speed and amount of HA on the BTCM surfaces are closely related to the calcium content in the BTCM matrix. Additionally, MTT assays showed that the BTCM samples are non-toxic to L929 cells, indicating that the BTCM samples have good biocompatibility. Cell adhesion experiments revealed that BTCM with higher CMA content can promote the attachment and proliferation of L929 cells. Thus, BTCM can simulate the dynamic mineralization and demineralization processes that occur in the oral cavity, making it a potential dental material capable of inducing the dynamic mineralization of HA in the oral environment. The novel CMA-based dental restorative resin showed remarkable bioactivity and biocompatibility, which makes it a promising dental restorative resin for use in dental restorative procedures and the field of biomedicine.

## Figures and Tables

**Table 1:** The formulation of the BTCM samples.

Sample	Bis-GMA (g)	TEGDMA (g)	CMA (g)	MAA (g)	CQ (g)	EDAB (g)
BTCM-1	7	3	0	0	0.1	0.1
BTCM-2	3.85	1.65	3	1.5	0.1	0.1
BTCM-3	2.8	1.2	4	2	0.1	0.1
BTCM-4	1.75	0.75	5	2.5	0.1	0.1

**Table 2:** Double bond conversion of BTCM.

Sample Code	DC/%		
	60 s	150 s	300 s
BTCM-1	87.12	93.72	96.79
BTCM-2	92.18	96.76	98.41
BTCM-3	95.58	98.21	98.59
BTCM-4	82.56	98.58	99.02

**Table 3:** The thermogravimetric analysis of the BTCM samples.

Sample Code	Residual Mass (%)
BTCM-1	4.67
BTCM-2	7.7
BTCM-3	10.1
BTCM-4	12.3

**Table 4:** The compression moduli of BTCM-1, BTCM-2, BTCM-3, and BTCM-4 after photopolymerization.

Sample Code	Compression Modulus
	Mean ± Standard Deviation (MPa)
BTCM-1	905.36 ± 10.68
BTCM-2	701.58 ± 20.13 ***
BTCM-3	625.71 ± 25.69 ***
BTCM-4	537.43 ± 23.45 ***

*** BTCM-2, BTCM-3, and BTCM-4 showed a statistically significant difference from BTCM-1, *p* = 0.000 < 0.005. *** *p* < 0.001.

**Table 5:** The OD values measured for L929 cells cultivated on BTCM samples at 24 h, 48 h, and 72 h, respectively.

Sample Code	OD Values (Mean ± Standard Deviation)		
	24 h	48 h	72 h
BTCM-1	0.863 ± 0.021	0.936 ± 0.046	0.911 ± 0.024
BTCM-2	0.888 ± 0.033	0.959 ± 0.042	0.902 ± 0.041
BTCM-3	0.886 ± 0.023	0.965 ± 0.049	0.948 ± 0.048
BTCM-4	0.888 ± 0.030	0.954 ± 0.044	0.955 ± 0.052
Positive control	0.883 ± 0.027	0.942 ± 0.045	0.985 ± 0.034
Negative control	0.057 ± 0.03 ***	0.054 ± 0.009 ***	0.034 ± 0.0218 ***

BTCM-1, BTCM-2, BTCM-3, BTCM-4, and positive control showed a statistically significance difference from the negative control, *p* = 0.000 < 0.005. *** *p* < 0.001.

## Data Availability

Not applicable.
